# Carotid blowout syndrome after nasopharyngeal carcinoma radiotherapy: successful treatment by internal carotid artery occlusion after stent implantation failure

**DOI:** 10.1186/s40064-016-3209-y

**Published:** 2016-09-13

**Authors:** Fei Dong, Qian Li, JianJun Wu, MinMing Zhang, GuangQiang Zhang, Bin Li, Kai Jin, Jie Min, WeiRen Liang, Ming Chao

**Affiliations:** Department of Radiology, The Second Affiliated Hospital, Zhejiang University School of Medicine, Hangzhou, 310009 China

**Keywords:** Carotid blowout syndrome, Endovascular treatment, Radiotherapy, Internal carotid artery

## Abstract

**Introduction:**

Carotid blowout syndrome (CBS) secondary to radiation therapy is life-threatening and requires emergency treatment. More recently, endovascular treatment has provided an effective way to control CBS-related bleeding.

**Case description:**

We present a case of CBS with a rupture of the internal carotid artery (ICA) pseudo-aneurysm after Gamma Knife radiation therapy for nasopharyngeal carcinoma (NPC). The patient was a 55-year-old man who was transferred to our hospital with severe repetitive epistaxis. He had a history of NPC and had been treated with Gamma Knife radiation therapy 7 months prior, with a central dose of 32 Gy and marginal dose of 16 Gy. As CBS was confirmed by angiography, and the affected part of the ICA lumen exhibited moderate stenosis, the patient was successfully treated by ICA occlusion after stent implantation failure. The patient died 40 months after this operation from tumor recurrence, but without epistaxis during follow up.

**Discussion and Evaluation:**

Quick selection of an appropriate treatment method is very important for an acute CBS patient.

**Conclusion:**

ICA occlusion can be directly considered for an acute CBS patient, if the affected ICA exhibits stenosis that is moderate or above.

## Background

Carotid blowout syndrome (CBS) is an uncommon, life-threatening complication in patients with radiation therapy for nasopharyngeal carcinoma (NPC) that requires emergency treatment. More recently, endovascular treatment has provided an effective way to control the bleeding associated with carotid blowout syndrome. In previous works, some researchers have suggested that stent placement is safe and feasible for this disease (Hakime et al. 2013; Farivar et al. 2014), and some argued that endovascular embolization provides both safe and effective management (He et al. 2013). Additionally, some researchers have found that there was no significant difference in the technical and hemostatic outcomes of stent placement and endovascular embolization (Chang et al. 2008). Here, we report a case of CBS secondary to Gamma Knife radiation therapy for nasopharyngeal carcinoma that was successfully treated by ICA occlusion after stent implantation failure.

## Case report

This study was approved by the Institutional Review Board and conformed with the principles outlined in the Declaration of Helsinki. A 55-year-old man was transferred to our hospital with severe repetitive epistaxis that had been occurring for 16 h. He had a history of NPC and had been treated with Gamma Knife radiotherapy 7 months prior. The central dose was 32 Gy, and the marginal dose was 16 Gy. He had no history of head and neck trauma. On admission to our hospital, physical examination showed a blood pressure of 106/78 mmHg, pulse rate of 150 beats per minute, and nasal bleeding. Blood test results were normal, except values for hemoglobin (Hb) (7.7 g/dL), platelet count (114,000/μL), prothrombin time (14.7 s), and plasma fibrinogen (1.2 g/L). Despite performing anterior and posterior nasal packing, approximately 400 mL of blood discharged from his mouth and nasal cavity 2 h later. Wadding was compressed, but massive oral and nasal bleeding emerged again 8 h later. His blood pressure dropped to 80/45 mmHg and pulse rate rose to 160 beats per minute. The patient was stabilized by continuous oxygen and massive intravenous serum infusion.

**Fig. 1:**
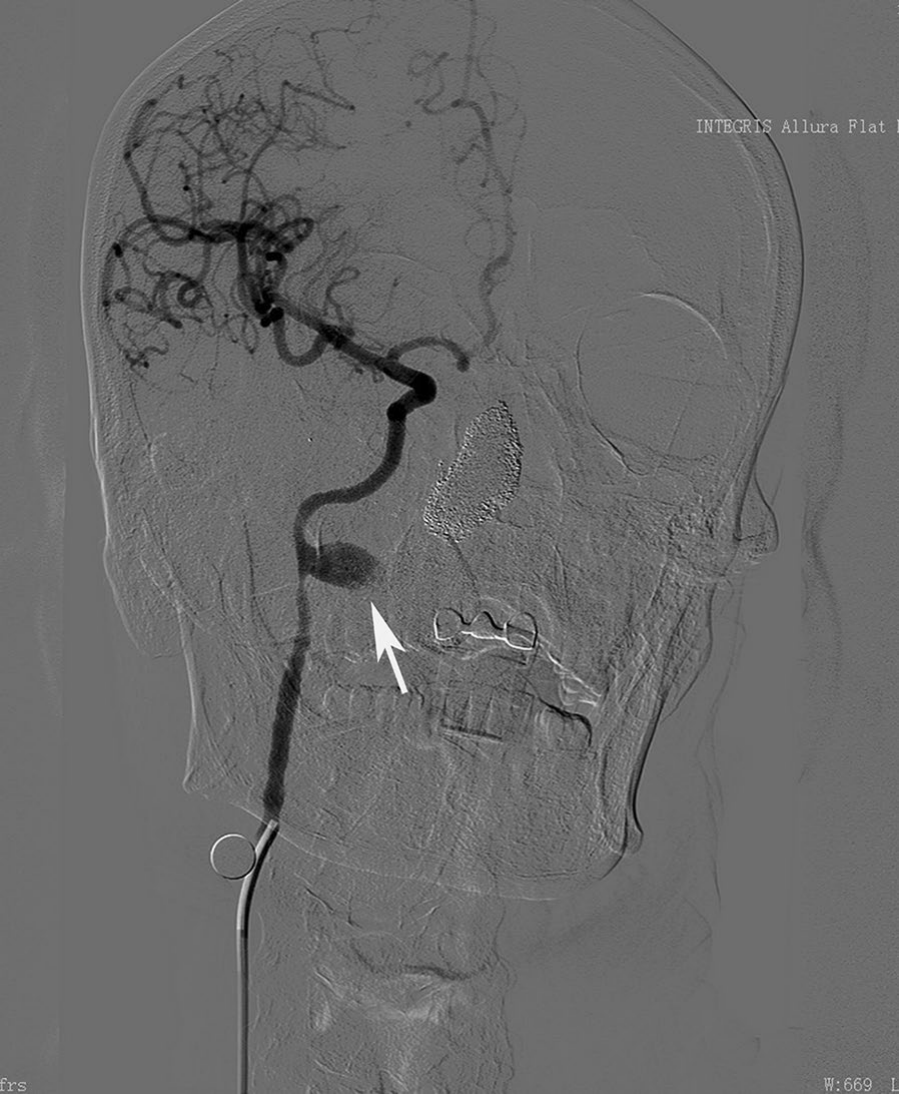
The angiogram shows a pseudo-aneurysm (*white arrow*) in the middle part of the right internal carotid artery at the nasopharyngeal level with the adjacent parent vessel, exhibiting moderate stenosis

**Fig. 2:**
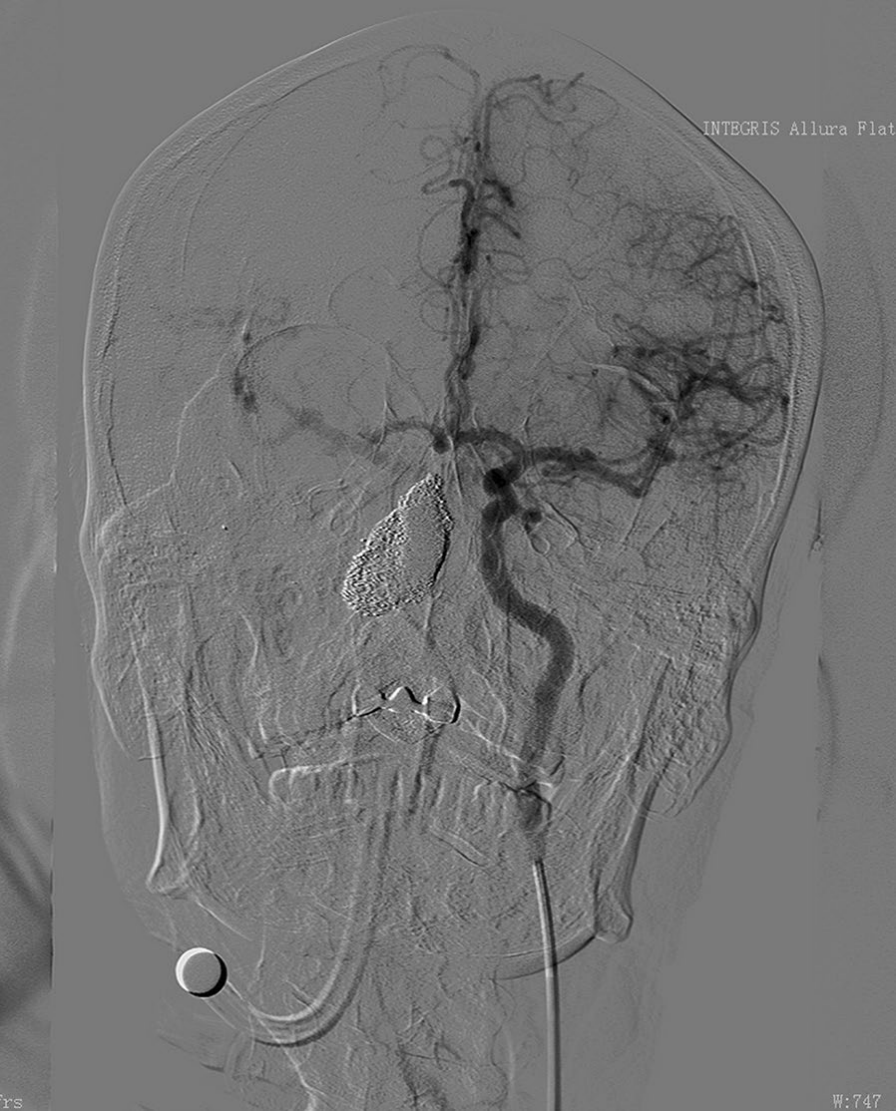
The angiogram shows good contralateral compensatory blood flow from the Circle of Willis when performing the compression test of the right ICA

**Fig. 3:**
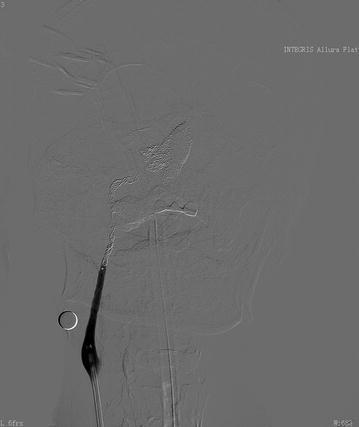
The angiogram shows that the pseudo-aneurysm disappears after ICA occlusion with microcoils, and there is no sign of bleeding

As CBS was suspected, digital subtraction angiography was performed. A pseudo-aneurysm approximately 17 mm × 10 mm in size was identified in the middle portion of the right internal carotid artery at the nasopharyngeal level, with adjacent parent vessel stenosis (Fig. 1). As the bleeding was repetitive and massive, we decided to use endovascular treatment. An emergency ICA compression test was performed, and it showed good compensatory blood flow from the Circle of Willis (Fig. 2). We then tried to implant a covered stent, which failed because of the narrow lumen. Massive bleeding occurred during the procedure. We immediately occluded the right ICA with coils, and the pseudo-aneurysm disappeared with the bleeding controlled (Fig. 3).

After ICA occlusion, the vital signs of the patient were stable. However, blood pressure began gradually dropping and ventricular escape rhythm occurred after 1 h. Symptomatic treatment was given, and he gradually recovered, though with some symptoms of paralysis. The patient died 40 months after this operation from tumor recurrence, but without epistaxis during follow up.

## Discussion

Carotid blowout syndrome (CBS) has been defined as the rupture of the carotid artery associated with hemorrhage or exposure of part of the carotid artery in a patient who has undergone aggressive management for head and neck cancer. Irradiation, radical surgery of head and neck tumors, and persistent or recurrent tumors are common predisposing factors for CBS (Luo et al. 2008; Yanik et al. 2011).

The formation mechanism of CBS after radiation therapy may be due to radiation-induced vascular injury, which may result from obliteration of the vasa vasorum, premature atherosclerosis, and weakening and necrosis of the arterial wall (Okamura et al. 2002). The mortality rate of carotid blowout after re-irradiation in head and neck cancer patients, according to a recent meta-analysis, was as high as 76 % (McDonald et al. 2012). It is reported that a total radiation dose to the neck ≥70 Gy is an independent risk factor associated with CBS (Chen et al. 2015). The radiation dose for our case was much less than 70 Gy. We speculate this risk may be due to the more intensive radiation with Gamma Knife than conventional radiotherapy.

Carotid blowout syndrome can present as one of three separate entities: threatened, impending, or acute carotid blowout (Cohen and Rad 2004). The present case was acute carotid blowout when admitted, and the bleeding was difficult to control by conventional methods. Thus, quick diagnosis and effective treatment are very important for this disease.

Angiography has been considered the gold standard for the diagnosis of CBS since the 1980s (Cohen and Rad 2004). We believe that once repetitive bleeding occurs in a patient with a head and neck neoplasm radiation history, carotid blowout syndrome should be suspected, and angiography should be performed as quickly as possible for further confirmation.

Emergency surgical ligation has traditionally been the only therapeutic maneuver available for CBS (Chaloupka et al. 1996). The mortality and morbidity rates of surgical ligation for acute hemorrhage patients are very high and can reach 40 and 60 %, respectively (Chaloupka et al. 1996).

More recently, endovascular treatment has provided an effective way to control the bleeding associated with carotid blowout syndrome. This method mainly includes two approaches; one is embolizing the vessel with detachable balloons or coils, and the other is preserving the vessel by stent, to cover the affected wall (Farivar et al. 2014).

Using covered stents for reconstruction of the affected ICA, at least in theory, is a more desirable treatment option, preserving patency of the carotid artery. However, stents cannot be successfully implanted in all patients (Haas and Ahn 2013). For the present case, stent implantation was initially chosen but failed because of moderate lumen stenosis and the emergent massive bleeding that occurred during the procedure due to the weakened vascular structure. We thought that the degree of lumen stenosis of the affected part of the ICA might be an important factor affecting stent implantation. If the lumen stenosis is moderate or above, stent implantation may be difficult.

Endovascular embolization allows occlusion of the affected ICA and is advantageous over stent implantation in its low rates of technical complications and rebleeding (Chang et al. 2015). However, embolizing the ICA may lead to severe cerebrovascular events. To ensure patient safety, performing a balloon test occlusion (BTO) is advocated prior to sacrificing the vessel (Haas and Ahn 2013). However, BTO is not always reliable. In a review of 8 years experience literature, 20 % (1 out of 5) of patients who passed the BTO had a cerebrovascular event (transient ischemic attack) after the ICA occlusion (Wan et al. 2013), and the probability of a cerebrovascular event is equal to those patients who were excluded from the BTO (1 out of 5, with left hemiplegia). Some authors have even proposed that in certain patients, BTO may be bypassed due to the emergency of the clinical situation (Haas and Ahn 2013). Our patient was in an emergency situation; the balloon test occlusion was not possible and only a carotid artery compression test was used as a substitute, but it too may be unreliable.

Despite all efforts, our case suffered transient cerebral ischemia 1 h after the operation. We believe that this event was associated with hypovolemia, vasospasm alleviation and compensatory insufficiency, which led to the dysfunction of cerebral autoregulation. Therefore, careful observation and symptomatic treatment to maintain stable systemic blood pressure is very important.

## Conclusions

In conclusion, carotid blowout syndrome secondary to radiation therapy is rare but life-threatening. Compared to conventional radiotherapy, a lower radiation dose may induce CBS in patients with Gamma Knife treatment for NPC. ICA occlusion can be directly considered if the affected ICA stenosis is moderate or above. Maintaining systemic blood pressure should be an important step to prevent ischemic complications after unilateral ICA occlusion.
